# XTP8 Promotes Ovarian Cancer Progression by Activating AKT/AMPK/mTOR Pathway to Regulate EMT

**DOI:** 10.1007/s12013-024-01246-4

**Published:** 2024-03-13

**Authors:** Ruixue Zhao, Xin Ning, Hongping Lu, Wei Xu, Jiaxin Ma, Jun Cheng, Rong Ma

**Affiliations:** 1 Department of Gynaecology, Harbin Medical University Cancer Hospital, Harbin, 150081 Heilongjiang China; 2 Hebei Utu Pharmaceutical Company Ltd, Shijiazhuang, Hebei Province 052165 China

**Keywords:** OC, XTP8, AKT/AMPK/mTOR, CALD1, EMT, Phenotype

## Abstract

Ovarian cancer (OC) ranks as the fifth leading cause of cancer-related death in women. The main contributors to the poor prognosis of ovarian cancer are the high rates of recurrence and metastasis. Studies have indicated a crucial role for hepatitis B virus X Ag-Transactivated Protein 8 (XTP8), a protein containing the DEP domain, in various cellular processes, including cell growth, movement, and differentiation, across several types of cancers. However, the role of XTP8 in ovarian cancer remains unclear. We observed elevated expression of XTP8 in ovarian cancer. Silencing XTP8 inhibited cell proliferation, promoted apoptosis, and yielded contrasting results in cells overexpressing XTP8. Furthermore, XTP8 facilitated ovarian cancer invasion and migration, triggering epithelial-mesenchymal transition (EMT). Mechanistically, XTP8 silencing led to reduced phosphorylation levels of AKT, increased p-AMPK levels, and decreased p-mTOR levels, while XTP8 overexpression exerted the opposite effects. Additionally, the activation of p-AMPK rescued the promoting effect of XTP8 on EMT in ovarian cancer cell lines, indicating that XTP8 acts as an oncogene by modulating the AKT/AMPK/mTOR pathway. Through transcriptome sequencing to identify downstream targets of XTP8, we found that XTP8 influences the expression of Caldesmon (CALD1) at both transcriptional and translational levels. CALD1 can be considered a downstream target of XTP8. The collaborative action of XTP8 and CALD1 activates the AKT/AMPK/mTOR pathway, regulating EMT to promote ovarian cancer progression. Inhibiting this signaling axis might represent a potential therapeutic target for ovarian cancer.

## Introduction

Ovarian cancer (OC) holds the highest mortality rate among all gynecologic malignancies. In the United States alone, it was estimated that there were approximately 19,880 new cases of OC, resulting in about 12,810 deaths in the year 2022 [1]. The principal factor contributing to the elevated mortality rate of ovarian cancer is its propensity for widespread metastasis, easily disseminating to adjacent organs through the peritoneum. Due to the small size and deep-seated location of the ovary within the abdominal cavity, early symptoms of ovarian cancer are subtle and lack specificity. Screening effectiveness is consequently limited, rendering early diagnosis challenging [2]. Despite notable progress in ovarian cancer treatment over the years, it persists as the fifth leading cause of cancer-related death in women [3]. Consequently, there is an escalating urgency in the quest for potential biomarkers to enable early diagnosis and facilitate timely intervention.

EMT is a dynamic process closely associated with cancer, wherein epithelial cells can transit along the E spectrum to the M spectrum, transforming from an epithelial to a mesenchymal phenotype [4]. One of the challenging aspects in the treatment of ovarian cancer is its unique mode of dissemination, distinct from the typical hematogenous invasion observed in other malignant tumors. Ovarian cancer follows a distinctive intraperitoneal spread pathway, characterized by direct extension and expansion of cancer cells [5]. After detachment from the primary tumor and entry into the peritoneal cavity, these cells either freely float in ascites or form multicellular aggregates (MCAs), which, upon interaction with the peritoneal cavity surface and the subepithelial extracellular matrix, give rise to secondary tumor lesions [6]. Throughout the EMT process, epithelial-type cancer cells undergo changes such as the loss of E-cadherin (E-cad) and an increase in N-cadherin (N-cad), leading to disruptions in cell-cell adhesion, loss of cell polarity, and remodeling of the cellular cytoskeleton, ultimately acquiring a mesenchymal phenotype [7]. These alterations are associated with changes in migratory and invasive characteristics.

The hepatitis B virus X Ag-Transactivated Protein 8 (XTP8) gene, also named DEP domain-containing protein 1B, located on human chromosome 5q12, encodes a 61 kDa protein consisting of 529 amino acids. XTP8 features an N-terminal DEP domain and a C-terminal RHO-GAP (GTPase activating protein)-like domain [8]. The DEP domain, a globular structure, primarily participates in signal transduction, determination of cell polarity, and mediation of plasma membrane anchoring [9]. The Rho-GAP domain regulates various biological activities, including its roles in actin polymerization, focal complex and focal adhesion assembly, cell cycle progression, membrane trafficking, cell adhesion, and cell polarity [10]. Functioning as an inhibitor of RhoA-based signal transduction complexes, XTP8 (A protein that accumulates in G2) exerts its effects by competitively interacting with PTPRF, promoting focal adhesion (FA) disassembly, and coordinating de-adhesion events and cell cycle progression during mitosis [11]. Research indicates that XTP8 is highly expressed in various tumors, playing a crucial role in tumor initiation and development. For instance, XTP8 was confirmed to be highly expressed in hepatocellular carcinoma, and promoted the proliferation and inhibited apoptosis of hepatocellular carcinoma cells by up-regulating the expression of FOXM1 [12]. XTP8 promotes the development of cholangiocarcinoma by enhancing the stability of CDK1 and regulating malignant phenotypes [13]. Additionally, XTP8 collaborates with GABRD to regulate the proliferation, apoptosis, invasion, and metastasis of esophageal squamous cell carcinoma [14]. However, the specific role and mechanism of XTP8 in ovarian cancer remain unclear.

In this study, immunohistochemical experiments based on clinical tissue were employed to reveal the expression levels of XTP8 in ovarian cancer. The correlation between XTP8 and overall survival (OS) time of ovarian cancer patients was investigated through the KM plotter database. Furthermore, the silencing and overexpression of XTP8 in ovarian cancer cells were achieved using small hairpin RNA (shRNA) and DNA plasmids, respectively, followed by a series of functional validations. The study also explored the relevant signaling pathways and downstream target genes regulated by XTP8 in the progression of ovarian cancer. In summary, the role of XTP8 in ovarian cancer was investigated at clinical, in vitro, and in vivo levels.

## Methods

### Cell Lines and Cell Culture

The human normal ovarian cell line IOSE80 was purchased from Wuhan Purdue Company, while the human ovarian cancer cell lines (A2780, OVCAR3, CAOV3, SKOV3, and ES-2) were obtained from the cell bank of the Chinese Academy of Sciences (Shanghai, China). IOSE-80, SKOV3, and CAOV3 cells were routinely cultured in DMEM medium (GIBCO, Grand Island, NY, USA) supplemented with 10% fetal bovine serum (FBS, GIBCO) and 1% penicillin/streptomycin (P/S). A2780, SKOV3 cells were maintained in RPMI1640 (GIBCO, Grand Island, NY, USA) medium supplemented with 10% FBS and 1% P/S. ES-2 cells were cultured in McCoy’s 5 A (GIBCO, Grand Island, NY, USA) containing 10% FBS and 1% P/S. All cells were cultured in 5% CO_2_ at 37 °C.

### Immunohistochemistry Analysis

Samples of ovarian cancer and normal ovarian tissue were collected from a total of 20 patients who underwent surgery at Harbin Medical University Cancer Hospital (Harbin, China) from March 2023 to July 2023. None of the patients had a history of other malignant tumors or had undergone radiotherapy or chemotherapy. Written informed consent was obtained from all patients for the use of their tissue samples for research. The research protocol has been approved by the Ethics Committee of Harbin Medical University (Harbin, China).

Paraffin-embedded sections were initially deparaffinized and rehydrated, followed by high-pressure antigen retrieval for 1 min using EDTA. After antigen retrieval, the sections were incubated overnight at 4 °C with the primary XTP8 antibody (1:200, PA5-72875, Invitrogen). Following washing with phosphate-buffered saline, sections were incubated with biotinylated anti-rabbit secondary antibody for 1 h at room temperature. Diaminobenzidine (DAB) staining was performed, and the slides were counterstained with hematoxylin. Microscopic images of the stained slides were captured. Two pathologists, blinded to clinical parameters, independently scored all tissues based on staining percentage and color intensity. Staining intensity was graded as follows: 0 (negative), 1 (weak), 2 (moderate), and 3 (strong). Staining percentage was classified as: 1 (1–24%), 2 (25–49%), 3 (50–74%), and 4 (75–100%). The product of the intensity and percentage scores was considered the final staining score for XTP8 (ranging from 0 to 12).

### RNA extraction and RT-qPCR

Total RNA from transfected cells was extracted using the Daktronics RNA Extraction Kit (8034111). Reverse transcription to cDNA was performed using a reverse transcription kit (RR037A, TaKaRa, Japan). The resulting cDNA was tested using a Real-time PCR kit (Power SYBR® Green PCR Master Mix, Invitrogen, USA). Primers were designed based on National Center for Biotechnology Information (NCBI) reference sequences and synthesized by Generaybio (Beijing, China). The primer sequences used are detailed in Supplementary Table 2. All primers were validated using a standard curve, ensuring amplification efficiencies were approximately 100%. The relative mRNA expression of target genes was calculated using the 2^-△△Ct^ method with β-actin as a control. All experiments were performed in triplicate.

### Western Blot Analysis

Transfected A2780 and OVCAR3 cells were lysed in a buffer containing 1% protein kinase inhibitor and 1% phosphokinase inhibitor to ensure complete cell dissolution. The cell lysate was then centrifuged at 12,000 × *g* for 15 min at 4 °C, and the supernatant was collected. Protein concentration was determined using the BCA method. Subsequently, loading buffer was added, and the samples were denatured at 100 °C for 10 min. Protein separation with 10% SDS-PAGE was performed, loading 60 μg per sample. The separated proteins were then transferred onto a PVDF membrane, which was blocked for 1 h at room temperature with 5% skim milk. The membrane was incubated with primary antibodies overnight at 4 °C, as listed in Table I of the Supplementary Material. After three washes with TBST, the membrane was incubated with secondary antibodies for 1 h at room temperature. The secondary antibody used was horseradish peroxidase-conjugated goat anti-rabbit (1:5000, ab6721, Abcam). The protein of interest was detected using Signal Fire ECL Reagent (6883, Cell Signaling, Technology) and quantified by densitometry with ImageJ software.

### Cell Transfection

Cell transfection was carried out using Polyplus transfection reagent (101000046). The lentiviruses containing shRNA targeting XTP8 (si-XTP8-1: 5’-CCUUGUUGGAGGAAGUCAUTT-3’; si-XTP8-2: 5’-GAUGCCGUUACGGAAACAUTT-3’; si-XTP8-3: 5’-CACAAGAGAACAUCCCAGUTT-3’). XTP8-overexpression (OE) and their corresponding scrambled control lentiviruses were purchased from Generaybio (Beijing, China). The density of cell transfection is 2 × 10^5^cells/well. Preparing the transfection system, jetPRIME buffer and siRNA or plasmid DNA were initially added, thoroughly shaken, and then jetPRIME reagent was added, followed by incubation at room temperature for 10 min. The prepared transfection reagent was slowly added to a 6-well plate, gently shake and mix, The prepared transfection reagent was slowly added to a 6-well plate. Stably transfected cells were screened with 3 μg/mL puromycin.

### Cell Viability Assay CCK8

OVCAR3 and A2780 cells were plated in 96-well plates and transfected after adherent cells. To prepare a CCK8 working solution, add 10 µL of CCK8 stock solution per 100 µL of medium. After 24 h of transfection, aspirate the medium, add 100 µL of CCK8 working solution to each well, re-put it into the incubator for 30 min, and then measure the absorbance at 450 nm with a microplate reader. Absorbance was measured 48 and 72 h after transfection, respectively.

### Cell Apoptosis

Prepare the caspase 3/7 reaction solution by thoroughly mixing caspase 3/7 buffer and the lyophilized caspase 3/7 foundation. The mixed solution can be stored at −20 °C in the dark after preparation. Thoroughly mix PBS and the caspase 3/7 reaction solution in a 1:1 ratio to obtain the working solution. After 72 h of cell transfection, the medium was aspirated, and 100 µL of Caspase 3/7 working solution was added to each well. The plates were protected from light, placed on a shaker at 300 rpm for 1 h. Aspirating 30 µL of working solution per well into a new 96-well plate for absorbance measurement.

### Transwell Assays

OVCAR3 and A2780 cells were transfected and cultured for 24 h, followed by a change in the culture medium to serum-free medium for a 12-h starvation treatment. The cells were then harvested, resuspended in serum-free medium, counted, and the cell concentration was adjusted to 1.5 × 10^5^/ml. For the Transwell migration assay, 30,000 cells per well were seeded in the upper chamber. The lower chamber of the Transwell was filled with 600 µL of medium containing 20% serum. The chamber was placed in the incubator, incubated for 36 h, and then removed. The upper chamber was washed twice with PBS, and the cells were fixed with anhydrous methanol for 20 min at room temperature. After discarding the methanol, the chamber was washed twice with PBS, followed by staining with 0.1% crystal violet for 10 min. The chamber was then washed three times with PBS. Cells on the inner side of the basement membrane were wiped off with a cotton swab. Microscopic images were captured at 100× magnification. The images were saved for subsequent counting and analysis.

### Wound-healing Assays

OVCAR3 and A2780 cells were seeded in 6-well plates and transfected 24 h after plating. Following 24 h of transfection and achieving confluency, a 10 μL pipette tip was used to create a straight scratch in the center of each well. Crossed cells were washed off with PBS, and serum-free medium was added. Photographs of the identical scratch locations were captured at 0 h and 48 h using a 100× field of view under the microscope.

### RNA Sequencing and GO Enrichment Analysis

Transient transfection was performed on OVCAR3 cells to silence or overexpress XTP8. Total RNA was extracted using Trizol, and mRNA-derived cDNA was sequenced on the Illumina platform for transcriptome analysis, yielding a substantial number of base sequences (Reads). The obtained Reads were finely filtered to obtain effective and high-quality Clean reads. These Clean reads were aligned to the Silva database using bowtie2 software to eliminate rRNA, and the remaining Reads were utilized for subsequent analyses. FeatureCount was applied for gene-level quantification, with results merged into a gene expression matrix for all samples. Gene differential expression significance analysis was conducted using EdgeR, resulting in a list of differentially expressed genes. TopGO software conducted GO enrichment analysis on the differentially expressed genes, counting the genes significantly enriched in each GO term, performing secondary classification statistics, and presenting the GO enrichment results through a scatter plot.

### Nude Mouse Models of Subcutaneous Tumorigenesis

Four-week-old female BALB/c nude mice (Charles River Laboratories, Wilmington, MA, USA) were procured for the experiment. All animal experiments were conducted in accordance with the Guidelines for the Care and Use of Laboratory Animals (NIH Publication Nos. 80-23, revised 1996) and the protocol approved by Harbin Medical University Cancer Hospital. OVCAR3 cells were employed to establish stable overexpression of XTP8 and control cell line. The cell concentration was adjusted to 10^7^ cells/ml. A 100 μL cell suspension was inoculated into the right axillary region of nude mice to establish two experimental groups: OE-Scramble and OE-XTP8 (*n* = 6 per group). Upon visible tumor formation, tumor volumes were measured every 3 days using calipers. Tumor volume is calculated as tumor volume (mm^3^) = A × B^2^×0.5, where A is the longest diameter and B is the shortest diameter. After 28 days post cell inoculation, the mice were euthanized. Subcutaneous tumors were dissected, recorded, and weighed. The tumor tissues, after snap-freezing in liquid nitrogen, can be stored at −80 °C.

### Statistical Analysis

The data are expressed as mean ± standard deviation. Each experiment was conducted in triplicate. The *t* test was employed for intergroup comparisons, and statistical significance was defined as *P* ≤ 0.05. Statistical analysis was performed using GraphPad Prism 5.0.2 (GraphPad, San Diego, CA, USA).

## Results

### Aberrant Expression of XTP8 in Ovarian Cancer and Its Prognostic Significance

**Fig. 1:**
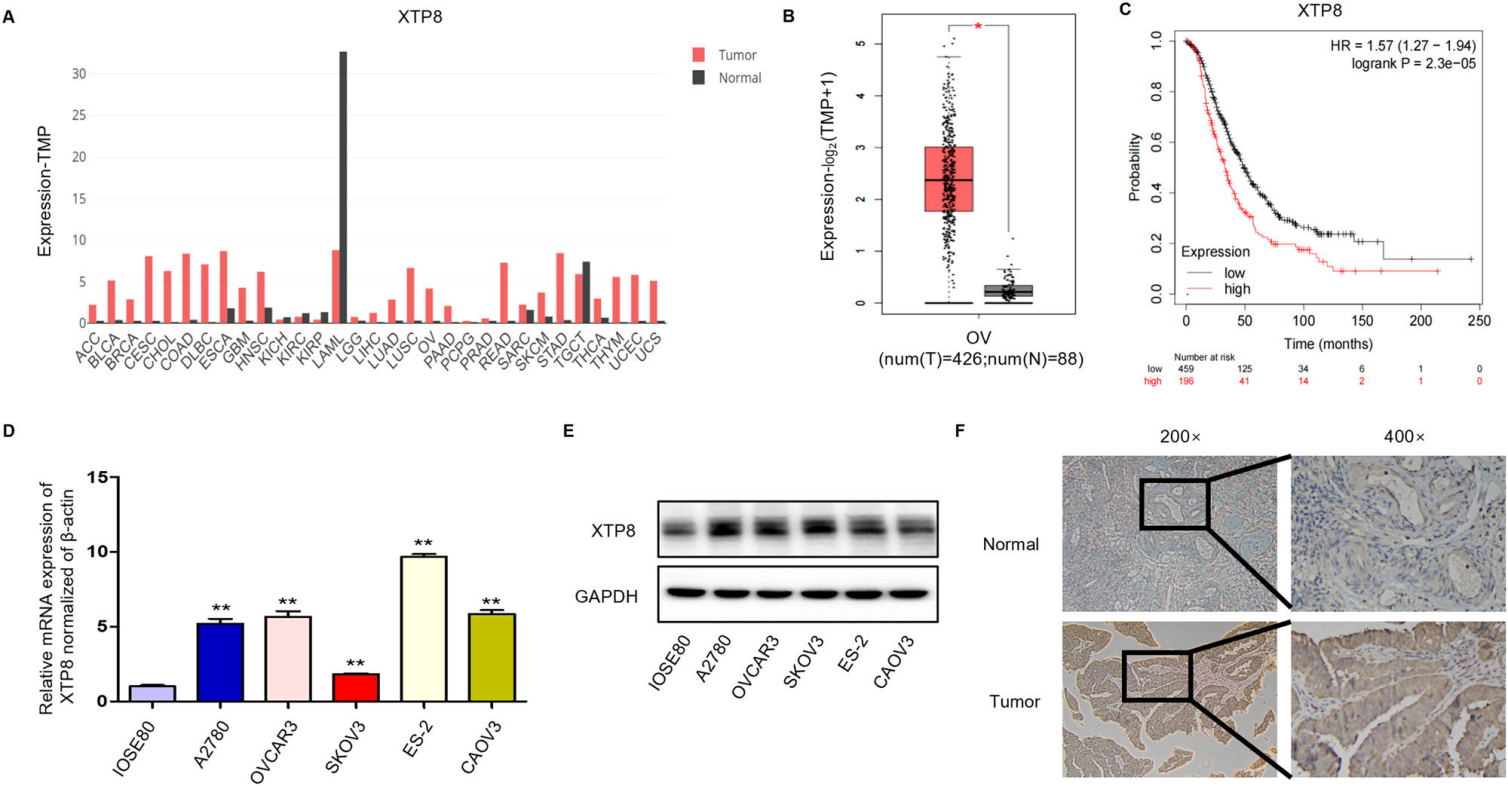
Expression of XTP8 in ovarian cancer. **A**, **B** XTP8 exhibits high expression in pan-cancer based on the GEPIA2 database, with elevated levels also observed in ovarian cancer. **C** Survival curve generated using KM PLOTTER reveals a negative correlation between XTP8 expression and overall survival (OS). **D**, **E** mRNA and protein expression levels of XTP8 in normal ovarian cell lines and five ovarian cancer cell lines (**p ≤ 0.01). **F** Elevated expression of XTP8 in ovarian serous adenocarcinoma tissues compared to normal tissues (Tumor *n* = 12, Normal *n* = 8) (*p* ≤ 0.05)

In this study, we initially employed the GEPIA2 database, revealing a significant upregulation of XTP8 in cancer tissues compared to normal tissues (Fig. 1A). Similarly, we observed a similar phenomenon in ovarian cancer (Fig. 1B). Utilizing KM PLOTTER for survival curve analysis, we identified a noteworthy negative correlation between elevated XTP8 expression levels and diminished OS in patients (Fig. 1C). This disparity demonstrated statistical significance. Subsequently, we conducted qPCR and Western blotting experiments utilizing the normal ovarian cell line IOSE80 and five ovarian cancer cell lines. The results demonstrated a substantial increase in XTP8 expression across all five ovarian cancer cell lines (A2780, OVCAR3, CAOV3, SKOV3, and ES-2) compared to the normal ovarian epithelial cell line (Fig. 1D, E). Notably, ES-2 exhibited the highest expression, while SKOV3 displayed the lowest. Consequently, A2780 and OVCAR3 cell lines were selected for subsequent experiments. Through immunohistochemical analysis, we scrutinized the expression of XTP8 in ovarian cancer and normal ovarian tissues. We observed XTP8 was expressed in the cytoplasm (Fig. 1F). In comparison to normal ovarian tissues, XTP8 was significantly overexpressed in ovarian cancer tissues(*P* ≤ 0.05), and this disparity bore statistical significance.

### XTP8 Promotes Ovarian Cancer Proliferation and Suppresses Apoptosis

**Fig. 2:**
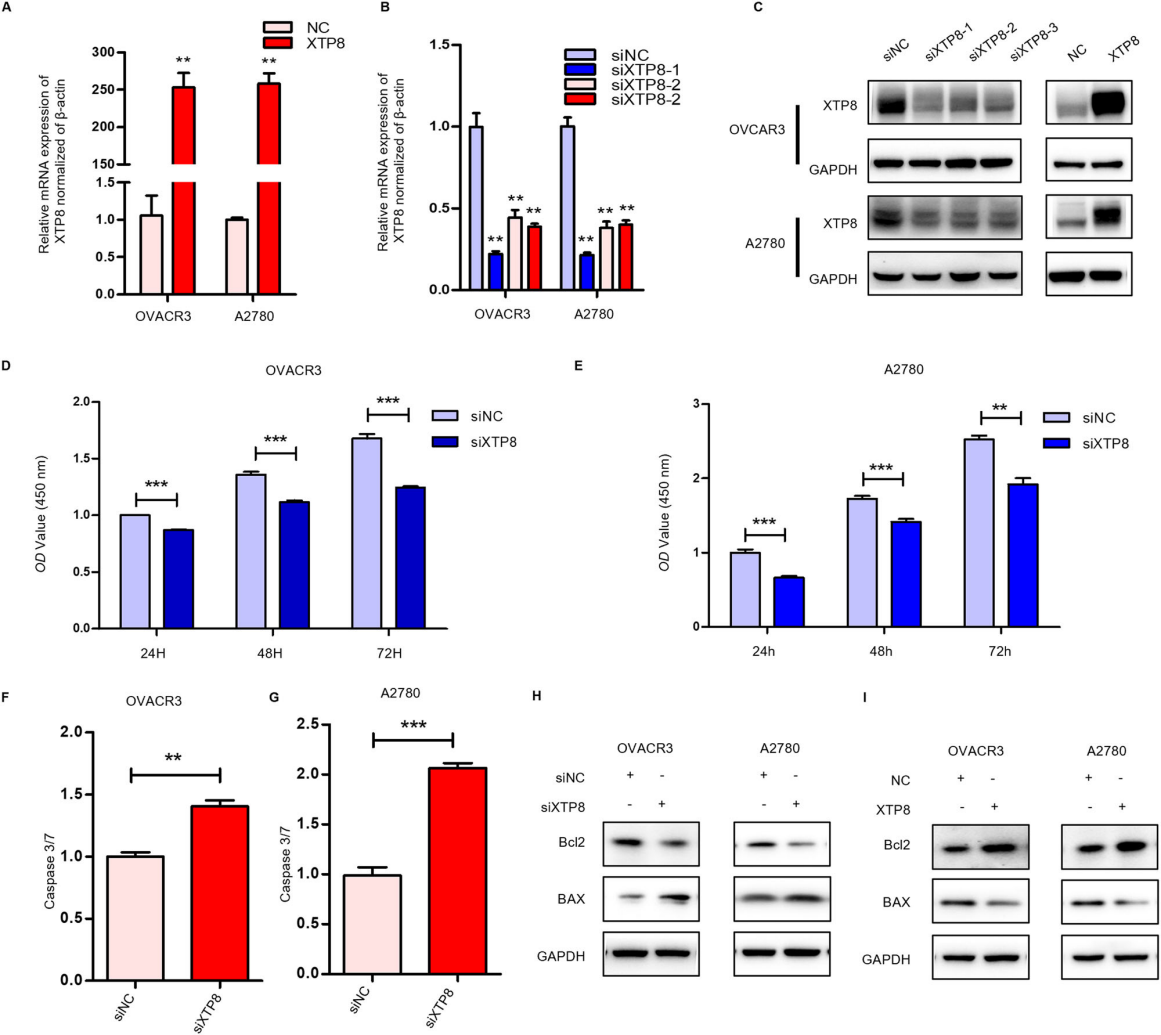
XTP8 promotes proliferation and inhibits apoptosis in OVCAR3 and A2780. **A**, **C** Plasmid effectively overexpresses XTP8 in OVCAR3 and A2780 cells. **B**, **C** siRNA effectively suppresses XTP8 expression in OVCAR3 and A2780. **D**, **E** Significant inhibition of ovarian cancer cell proliferation upon XTP8 knockdown. **F**, **G** Marked promotion of ovarian cancer cell apoptosis upon XTP8 knockdown. **H**, **I** XTP8 affects ovarian cancer cell apoptosis through BCL2/BAX modulation. (*p ≤ 0.05, **p ≤ 0.01, ***p ≤ 0.001)

We designed three siRNA targeting XTP8 and synthesized plasmids for overexpressing XTP8 and transfected them into ovarian cancer cell lines OVCAR3 and A2780. Subsequently, we conducted RT-PCR and Western blot to assess the silencing and overexpression effects. As depicted in Fig. 2A–C, the overexpression was significant, and siXTP8-1 exhibiting the best silencing effect in both OVCAR3 and A2780 cell lines. Therefore, siXTP8-1 was chosen for subsequent experiments.

CCK8 experiments were employed to determine the role of XTP8 in the proliferation of OVCAR3 and A2780 cells. The experimental results showed in Fig. 2D, E, compared with the control group, the proliferation ability of OVCCR3 and A2780 cells was significantly weakened after XTP8 interference and enhanced after XTP8 overexpression (Supplementary Fig. 1A, B). Additionally, Caspase3/7 experiments were conducted, revealing increased relative activity of Caspase3/7 in OVCAR3 and A2780 cells after XTP8 interference and decreased activity after XTP8 overexpression (Fig. 2F, G, Supplementary Fig. 1C, D). Subsequent Western Blot analysis of apoptosis-related markers showed that overexpression of XTP8 increased BCL2 expression and decreased BAX expression, and knockdown of XTP8 decreased BCL2 expression and increased BAX expression in OVCAR3 and A2780 cells (Fig. 2H, I). In summary, our experimental results suggest that XTP8 promotes ovarian cancer proliferation and inhibits apoptosis. Moreover, XTP8 exerts its influence on the apoptosis of ovarian cancer cells through the BCL2/BAX pathway.

### XTP8 promotes the invasion and migration of ovarian cancer cells in vitro

**Fig. 3:**
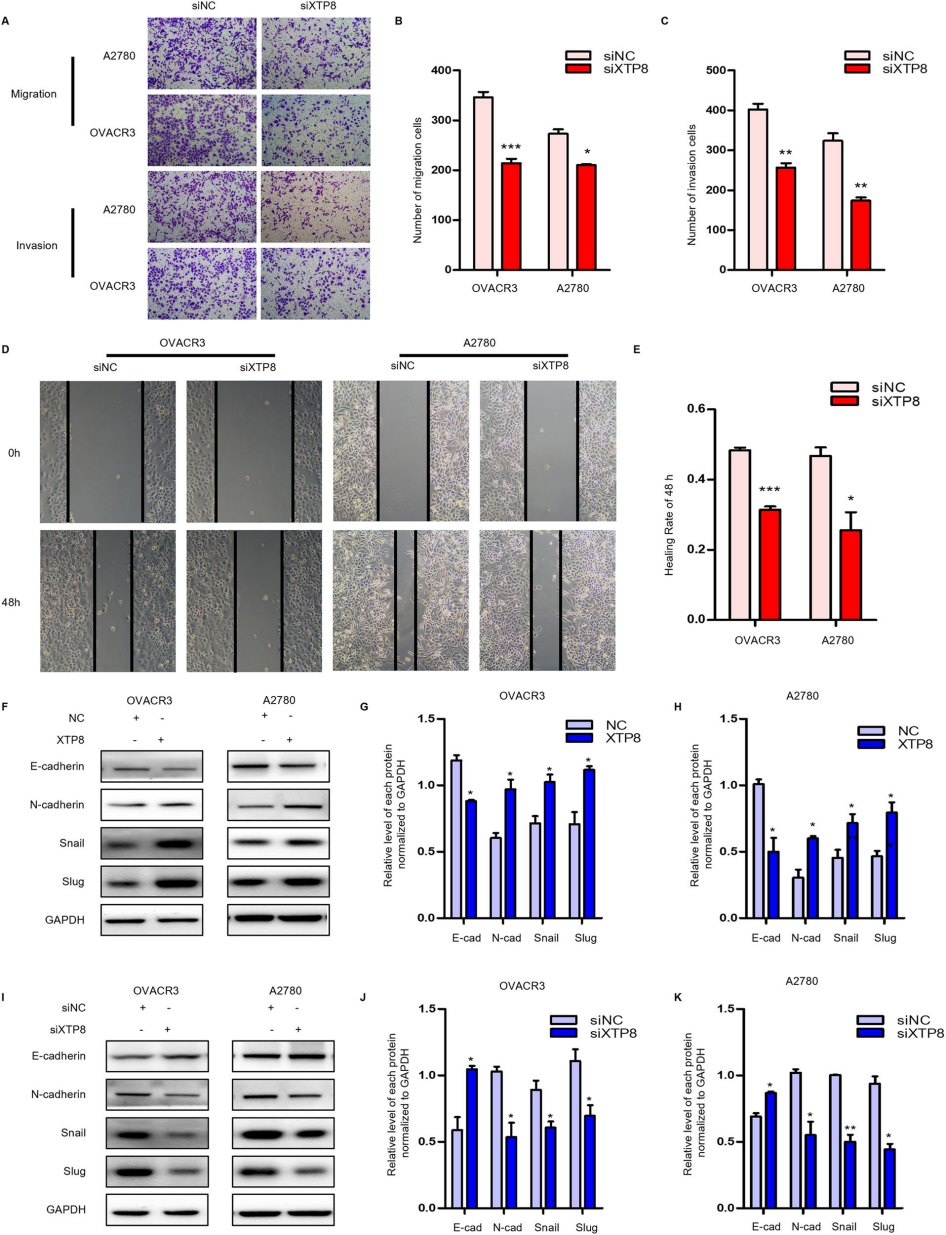
XTP8 promotes ovarian cancer metastasis, invasion, and EMT. **A**–**C** Transwell assays reveal a significant decrease in the number of migrated and invaded cells after XTP8 silencing. **D**, **E** Scratch assays demonstrate marked inhibition of wound healing upon XTP8 knockdown. **F**–**H** Overexpression of XTP8 induces a mesenchymal phenotype, with a decrease in E-cadherin expression and an increase in N-cadherin, Snail, and Slug expression. **I**–**K** Silencing XTP8 results in an increase in E-cadherin expression and a decrease in N-cadherin, Snail, and Slug expression. (*p ≤ 0.05, **p ≤ 0.01, ***p ≤ 0.001)

To investigate the impact of XTP8 on ovarian cancer cell invasion and migration, we conducted Transwell and scratch assays. Transwell migration and invasion assay showed that the number of invasion and migration of OVCAR3 and A2780 cells in si-XTP8 group was significantly lower than that in siNC group (Fig. 3A–C), which was opposite in XTP8 overexpression group (Supplementary Fig. 2A–C). As shown in the Fig. 3D, E, silencing XTP8 inhibited wound closure, whereas overexpression of XTP8 promoted wound closure at 48 h compared with the control group (Supplementary Fig. 2D, E). These results indicate that XTP8 promotes the prometastatic phenotype of ovarian cancer cells.

We conducted the expression levels of EMT-related proteins in OVCAR3 and A2780 cells after transfection through protein imprinting experiments to validate the contribution of XTP8 to EMT. As shown in the Fig. 3F–H, overexpression of XTP8 resulted in a mesenchymal phenotype, characterized by decreased expression of E-cadherin and increased expression of N-cadherin, Snail and Slug. Conversely, silencing XTP8 led to an epithelial phenotype, characterized by increased expression of E-cadherin and decreased expression of N-cadherin, Snail, and Slug (Fig. 3I–K). These differences were statistically significant. In summary, our results indicate that XTP8 promotes the invasion and migration of cells in ovarian cancer and plays a crucial role in triggering tumor EMT.

### XTP8 Modulates Ovarian Cancer Progression and EMT through the AKT/AMPK/mTOR Signaling Pathway

**Fig. 4:**
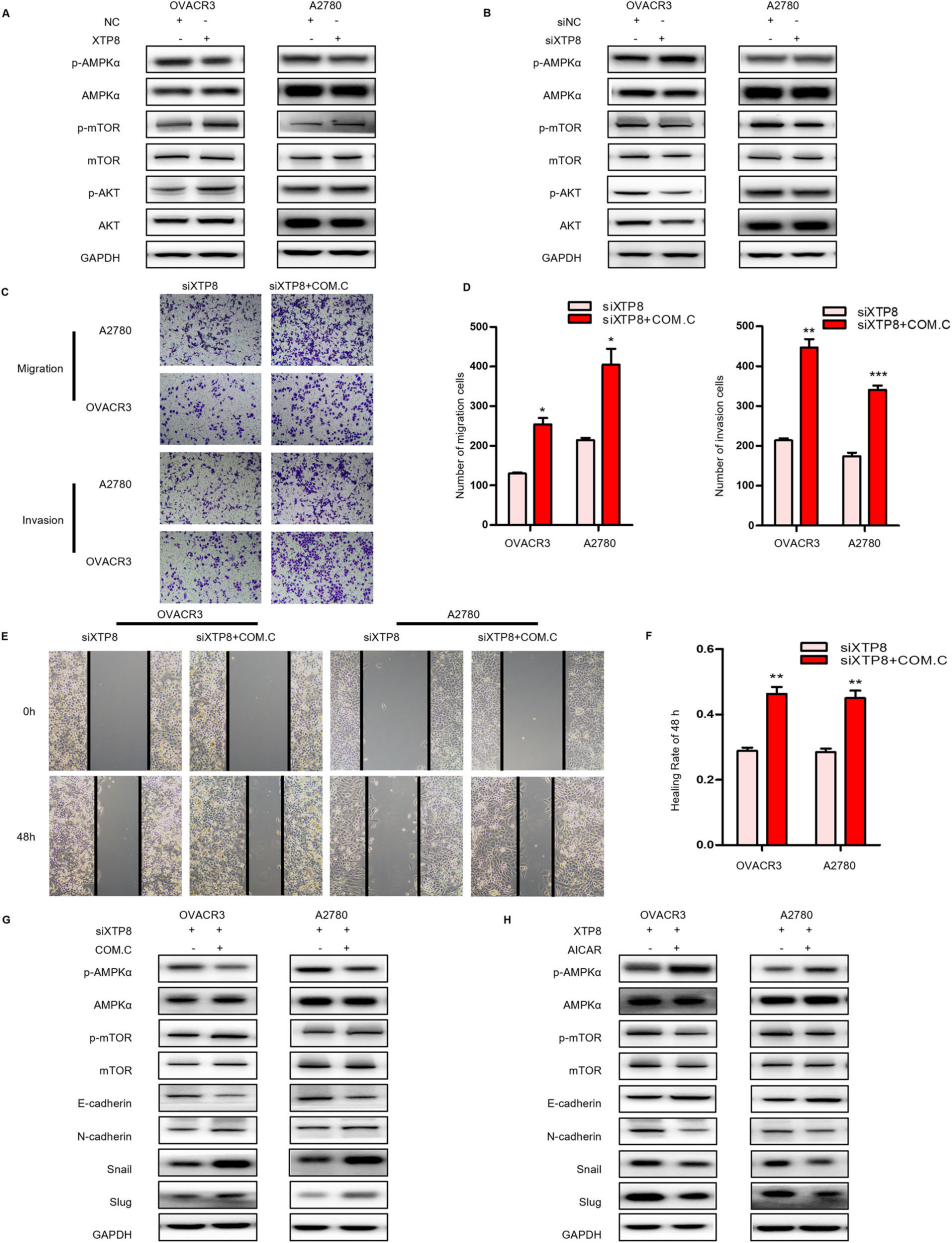
XTP8 influences ovarian cancer progression and EMT through the AKT/AMPK/mTOR signaling pathway. **A** Overexpression of XTP8 activates AKT/mTOR and inhibits AMPK activity. **B** Silencing XTP8 results in decreased phosphorylation of AKT, increased p-AMPK, and decreased p-mTOR levels. **C**, **D** Transwell assays demonstrate that an AMPK inhibitor reverses the decrease in invasive and migratory cells caused by XTP8 silencing. **E**, **F** Scratch assays reveal that an AMPK inhibitor reverses the slow wound healing caused by XTP8 silencing. **G** Treatment with an AMPK inhibitor reduces E-cadherin expression and increases N-cadherin, Snail, and Slug expression. **H** Treatment with an AMPK activator increases E-cadherin expression and suppresses N-cadherin, Snail, and Slug expression. (*p ≤ 0.05, **p ≤ 0.01, ***p ≤ 0.001)

In a study conducted by Wu et al., it was demonstrated that XTP8 potentially enhances the proliferation of EOC cells by promoting AKT phosphorylation at Ser473 [15]. We measured the protein expression levels of core components in the AMPK/AKT/mTOR pathway in OVCAR3 and A2780 cells after silencing and overexpressing XTP8. As illustrated in Fig. 4A, B, overexpression of XTP8 activated the AKT/mTOR pathway, inhibited AMPK activity, leading to increased expression of p-AMPK. Conversely, silencing XTP8 resulted in decreased phosphorylation levels of AKT, increased p-AMPK, and decreased p-mTOR.

COM.C functions as an inhibitor of AMPK activity. We simultaneously silenced XTP8 and treated OVCAR3 and A2780 cell lines with COM.C, followed by wound healing and Transwell assays. The results indicated that the AMPK activity inhibitor could reverse the inhibitory effect on migration and invasion in both cell lines after XTP8 silencing (Fig. 4C–F). In contrast, AICAR, an AMPK activator, was applied simultaneously with XTP8 overexpression in OVCAR3 and A2780 cell lines, and wound healing and Transwell assays were conducted. The results revealed that the AMPK activator could counteract the enhanced migratory and invasive properties induced by XTP8 overexpression (Supplementary Fig. 3A–D).To validate whether XTP8 affects tumor EMT through the AKT/AMPK/MTOR signaling pathway, we also examined the expression of EMT-related proteins. As illustrated in Fig. 4G, H, treatment with the AMPK activator increased E-cadherin expression and suppressed N-cadherin, Snail, and Slug expression. Conversely, treatment with the AMPK inhibitor reduced E-cadherin expression and increased N-cadherin, Snail, and Slug expression. These results suggest that XTP8 promotes ovarian cancer progression and EMT by activating the AKT/AMPK/MTOR pathway.

### CALD1 as a Downstream Target of XTP8

**Fig. 5:**
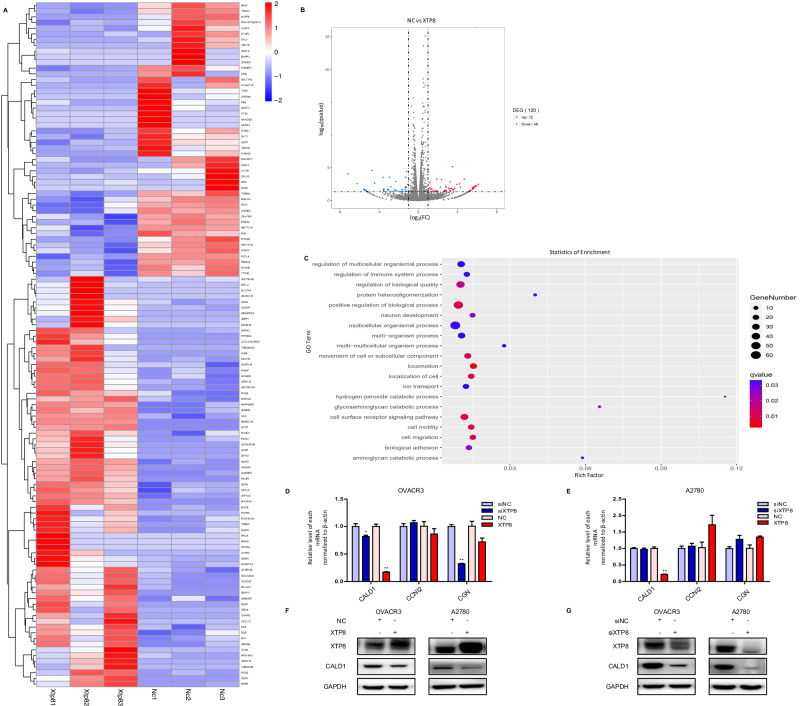
CALD1 as a downstream target of XTP8. **A**, **B** Compared to the control group, overexpression of XTP8 resulted in 120 differentially expressed genes, with 72 up-regulated and 48 down-regulated. **C** GO enrichment analysis of the 120 differentially expressed genes generated after overexpression of XTP8. **D**, **E** q-PCR validation of the expression levels of the 3 selected differentially expressed genes. **F**, **G** Both silencing and overexpression of XTP8 led to a decrease in CALD1 protein expression levels

OVCAR3 cells were transiently transfected to silence or overexpress XTP8, followed by RNA extraction for transcriptome sequencing. Analysis of the sequencing results revealed a total of 163 differentially expressed genes after silencing XTP8, with 86 upregulated and 77 downregulated (Supplementary Fig. 4A, B). Overexpression of XTP8 resulted in 120 differentially expressed genes, including 72 upregulated and 48 downregulated (Fig. 5A, B). GO enrichment analysis of the differentially expressed genes generated upon overexpression of XTP8 showed significant enrichment in biological processes related to cell interaction, such as cell migration, biological adhesion, cell surface receptor signaling pathway, and cellular activity Fig. 5C. This further validates the impact of XTP8 on biological processes involving cell interaction.

Among these differentially expressed genes, we selected six genes for further exploration based on a literature review and the magnitude of expression differences. However, three genes were not analytically quantifiable due to primer-related issues. The results for the remaining three genes are presented in the Fig. 5D, E, revealing that XTP8’s impact on their expression is unstable across different cell lines and RNA batches (Supplementary Fig. 4C). Notably, the reduction in CALD1 expression after XTP8 overexpression was consistent and significantly different. Consequently, CALD1 was selected for Western blot analysis. As illustrated in the Fig. 5F, G, in line with qPCR results, both XTP8 interference and overexpression led to a reduction in CALD1. These findings suggest that XTP8 can influence CALD1 expression at both the transcriptional and translational levels, indicating CALD1 as a downstream target of XTP8.

### Overexpression of XTP8 Promotes Tumor Growth In Vivo

**Fig. 6:**
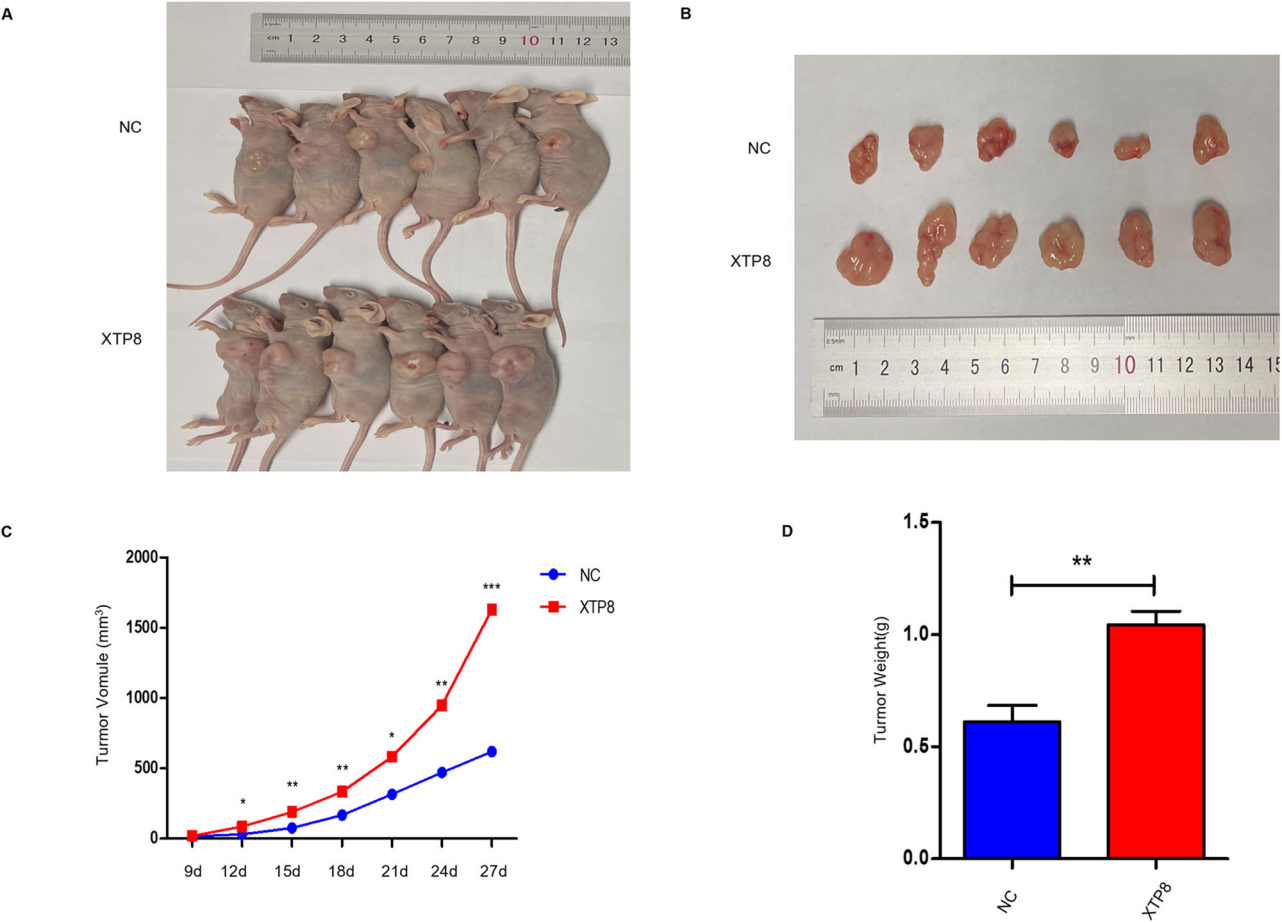
In vivo xenograft tumor model confirms that overexpression of XTP8 promotes tumor growth. **A**, **B** OVCAR3 cells transfected with NC or XTP8 were subcutaneously injected into the axilla of nude mice, and the volume of each tumor was measured at specific time points. **C** Growth curve of tumors, showing a significantly higher growth rate in mice treated with XTP8 compared to the control group. **D** Average tumor weight in mice treated with XTP8 is significantly higher than in the control group

Building upon the in vitro findings, we established an in vivo xenograft model to validate the role of XTP8 in ovarian cancer. Tumor formation was visually observed on the 9th day after implantation. Subsequently, tumor dimensions were measured using calipers on the 12th, 15th, 18th, 21st, 24th, and 27th days, and tumor volume was calculated using the respective formula. After 27 days of subcutaneous injection of OVCAR3 cells, mice were euthanized, and tumors were excised and weighed (Fig. 6A, B). The growth curve revealed a significant increase in tumor volume in mice injected with cells stably overexpressing XTP8 compared to the control group (Fig. 6C). The final tumor weight also exhibited a significant increase (Fig. 6D).

## Discussion

The treatment process for ovarian cancer typically involves initial surgery to achieve maximal tumor removal, followed by 6-8 cycles of chemotherapy postoperatively. Targeted therapy tailored to the patient’s Homologous Recombination Deficiency (HRD) status may also be administered. Despite these interventions, approximately 80% of patients experience recurrence within 5 years. For recurrent cases, where surgical options are limited, disease control primarily relies on chemotherapy. Repeated chemotherapy and the development of drug resistance significantly impact patients’ quality of life. Therefore, a major goal for prognosis improvement is to inhibit tumor recurrence, metastasis, and prevent drug resistance. In recent years, targeted therapy has become a focal point of research and clinical attention in cancer treatment.

Protein domains are fundamental units of protein structure and function, and many proteins contain one or more structural domains. The DEP domain is a protein motif consisting of approximately 100 amino acid residues [16]. While its primary function is membrane anchoring, it can exhibit other diverse functions through the use of different binding interfaces, such as signal transduction and determination of cell polarity [9]. Notably, DEP domain-containing protein 1 A (DEPDC1A), also known as DEPDC1, has been closely associated with the development and prognosis of human cancers. Studies suggest that DEPDC1A plays a oncogenic role in oral squamous cell carcinoma (OSCC) and can promote aerobic glycolysis, migration, and invasion through the Wnt/β-catenin pathway [17]. Another protein containing the DEP domain, XTP8, has been demonstrated to participate in various cellular processes, including cell growth, movement, and differentiation, playing a crucial role in multiple tumors [10]. For instance, downregulation of XTP8 activates the p53 pathway to halt the progression of multiple myeloma (MM), and CCNB1 has been identified as a downstream target interacting with the p53 pathway [18]. Research by Ouyang et al suggests that when XTP8 is downregulated in glioblastoma, the inhibitory effect of Platycodin D (PD) on tumor growth is enhanced, while overexpression of XTP8 reverses PD’s inhibitory effects on glioblastoma proliferation and migration [19]. Given the multifaceted roles of XTP8 in various tumors, we investigated its functions and mechanisms in ovarian cancer. Utilizing GEPIA2 and KM PLOTTER online databases and immunohistochemistry experiments, we revealed the expression levels of XTP8 in ovarian cancer, confirming its overexpression and association with OS. Through a series of in vitro experiments, we found that XTP8 promotes cell proliferation, inhibits apoptosis, and facilitates tumor migration and invasion in ovarian cancer.

Currently, numerous studies suggest that EMT is associated with tumor metastasis. Therefore, inhibiting EMT may be an effective therapeutic strategy for ovarian cancer, slowing down disease progression. In tumors, EMT can enhance the initiation of tumor-initiating cells and the migration of tumor cells, leading to resistance to various treatment modalities [20]. Activation of EMT can also impact the quantity and function of immune cells in the tumor microenvironment, with tumor cells expressing EMT-related markers exhibiting stronger resistance to both innate and adaptive immunity. For example, melanoma cells expressing Snail can secrete TGFβ, inducing the formation of Treg cells while inhibiting the antigen-presenting ability of dendritic cells. Reversing Snail expression can restore the immune response against tumors and increase sensitivity to immunotherapy [21]. In this study, we observed that silencing XTP8 suppressed the invasion and migration of ovarian cancer cells. There was an increase in the expression of E-cadherin, while the expression of N-cadherin, Snail, and Slug was downregulated. These findings indicate that after silencing XTP8, the mesenchymal phenotype shifted towards the epithelial phenotype, inhibiting EMT in ovarian cancer.

The Akt/AMPK/mTOR pathway is a crucial signaling pathway that regulates cell metabolism, energy homeostasis, and cell growth [22]. Akt regulates protein synthesis, cell proliferation, and differentiation by activating mTOR. Tumor cells and transformed cells with disrupted mTOR signaling are more sensitive to mTOR inhibitors than normal cells, making mTOR an important target for cancer treatment [23]. The Akt/AMPK/mTOR pathway is also a significant signaling pathway in the development and progression of tumors. MEDAG has been shown to regulate breast cancer (BC) progression and EMT via the AKT/AMPK/mTOR pathway, reducing chemosensitivity in BC cells [24]. SQSTM1/p62 promotes thyroid cancer cell growth and induces autophagy by modulating the AKT/AMPK/mTOR signaling pathway [25]. Resveratrol significantly inhibits the proliferation, migration, and invasion of A2780 and SKOV3 ovarian cancer cells through the AMPK/mTOR signaling pathway, while impairing glycolysis and inducing apoptosis in these cells [26]. Our experiments indicate that XTP8 activates the AKT/AMPK/mTOR pathway, enhancing migration, invasion, and EMT. The application of AMPK inhibitors or agonists partially reverses these effects, suggesting that XTP8 may influence ovarian cancer by modulating this pathway.

CALD1 is a cell cytoskeleton-associated protein primarily found in smooth muscle and non-smooth muscle cells [27]. CALD1 plays a role in the reorganization of the cell cytoskeleton through interaction with recombinant myosin, regulating cell proliferation, migration, and invasion [28]. Studies have identified CALD1 as an EMT transcription factor and a target of numerous genes regulating tumor EMT [29]. miR-1278 negatively regulates CALD1 expression, affecting the migration of gastric cancer cells by modulating the MAPK pathway [30]. CALD1 acts as a downstream target of AHSA1, promoting proliferation and EMT in liver cancer [31]. Moreover, CALD1 has been found to be associated with various immune cells and immune components in the tumor microenvironment. CALD1 expression correlates positively with immune cells such as CD8^+^ T cells, CD4^+^ T cells, macrophages, neutrophils, and others in gastric cancer [32]. Research by Li and colleagues suggests that CALD1 can upregulate PD-L1 expression through the JAK/STAT signaling pathway [33]. We conducted transcriptome sequencing on OVCAR3 cells following XTP8 interference, revealing differential expression of CALD1 among the genes. Subsequent validation at both RNA and protein levels confirmed a decrease in CALD1 expression upon XTP8 interference. Notably, both XTP8 interference and overexpression resulted in decreased CALD1 expression, with a more pronounced decrease observed at the transcriptional level in the overexpression group and a more significant reduction at the protein level in the interference group. This observation may be attributed to the intricate regulatory mechanisms among proteins. In conclusion, CALD1 serves as a downstream target of XTP8. The impact of CALD1 on the ovarian cancer phenotype and the specific regulatory mechanisms by which XTP8 modulates CALD1 represent avenues for future research.

In summary, our results elucidate the role of XTP8 in ovarian cancer and its underlying mechanisms. XTP8 exhibits high expression in ovarian cancer and is correlated with OS. XTP8 promotes ovarian cancer cell proliferation while inhibiting apoptosis through the BCL2/BAX pathway. Additionally, XTP8 enhances ovarian cancer migration, invasion, and EMT via the AKT/AMPK/mTOR signaling pathway. Simultaneously, it downregulates CALD1 expression at both the transcriptional and translational levels.

### Supplementary Information

Supplement material

western blot

## Data Availability

The datasets generated during and/or analyzed during the current study are available from the corresponding author on reasonable request.
