# Supplementary material for: XTP8 Promotes Ovarian Cancer Progression by Activating AKT/AMPK/mTOR Pathway to Regulate EMT
Source: Cell Biochem Biophys. 2024 Mar 13;82(2):945–57. doi: 10.1007/s12013-024-01246-4 (PMC11344704; doi:10.1007/s12013-024-01246-4)
Supplement: Supplementary file 1 — Supplement material [file 12013_2024_1246_MOESM1_ESM.docx]

| Table 1 Antibody Information | | | |
| --- | --- | --- | --- |
| Anti-DEPDC1B | 1:1000 | PA5-72875 | Invitrogen |
| Anti-BAX | 1:1000 | 5023S | Cell Signaling Technologies |
| Anti-E-cadherin | 1:1000 | 3195S | Cell Signaling Technologies |
| Anti-N-cadherin | 1:1000 | 13116S | Cell Signaling Technologies |
| Anti-Snail | 1:1000 | 3879T | Cell Signaling Technologies |
| Anti-Slug | 1:1000 | 9585T | Cell Signaling Technologies |
| Anti-p-AMPKα | 1:1000 | 2535S | Cell Signaling Technologies |
| Anti-AMPKα | 1:1000 | 5831S | Cell Signaling Technologies |
| Anti-p-mTOR | 1:1000 | 2971S | Cell Signaling Technologies |
| Anti-mTOR | 1:1000 | 2983S | Cell Signaling Technologies |
| Anti-p-AKT | 1:1000 | 4060S | Cell Signaling Technologies |
| Anti-AKT | 1:1000 | 4691S | Cell Signaling Technologies |
| Anti-CALD1 | 1:500 | ab32330 | Abcam |
| Anti-GAPDH | 1:1000 | 5174S | Cell Signaling Technologies |

| Table 2 Primer Information | | |
| --- | --- | --- |
| Name | Forward | Reverse |
| DEPDC1B | 5'-AGCTACCAGGCTGTGGAATG-3' | 5'-AGCTCTTGAAACGACAGCGA-3' |
| CALD1 | 5'-TGGAGGTGAATGCCCAGAAC-3' | 5'-GAAGGCGTTTTTGGCGTCTTT-3' |
| CCNI2 | 5'-CCAGGGAGTATGAATGAATGTT-3' | 5'-TTGGGATAAGCCTGGGAAGTT-3' |
| CGN | 5’-GAAGCGTTTGCTGGACAGGAC-3’ | 5’-TGCTGCAGGGCTTGCTTAGA-3’ |
| β-actin | 5‘’-CAGAGCCTCGCCTTTGCC-3‘ | 5‘-GTCGCCCACATAGGAATC-3‘ |

**Fig.S1**
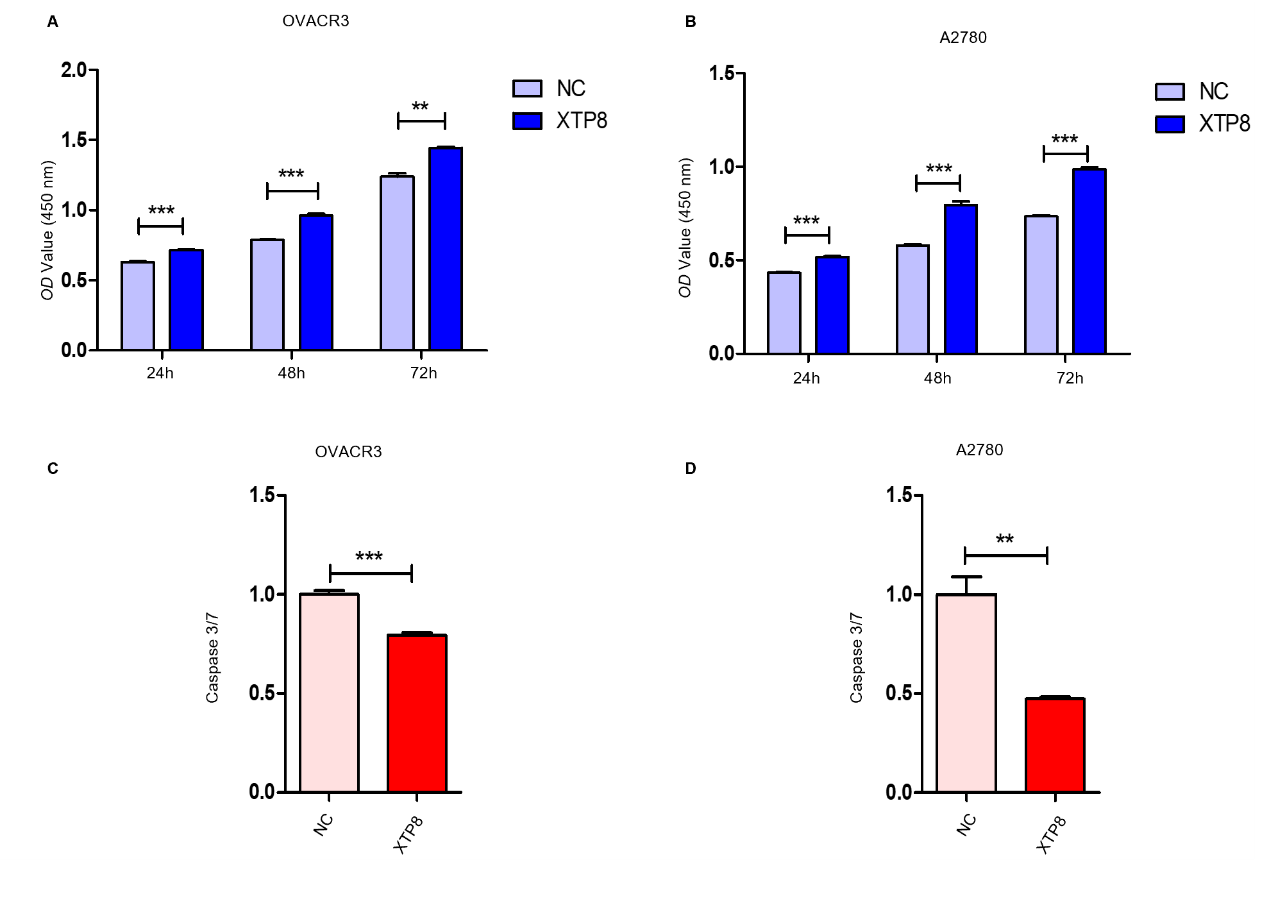


**Fig.S2**


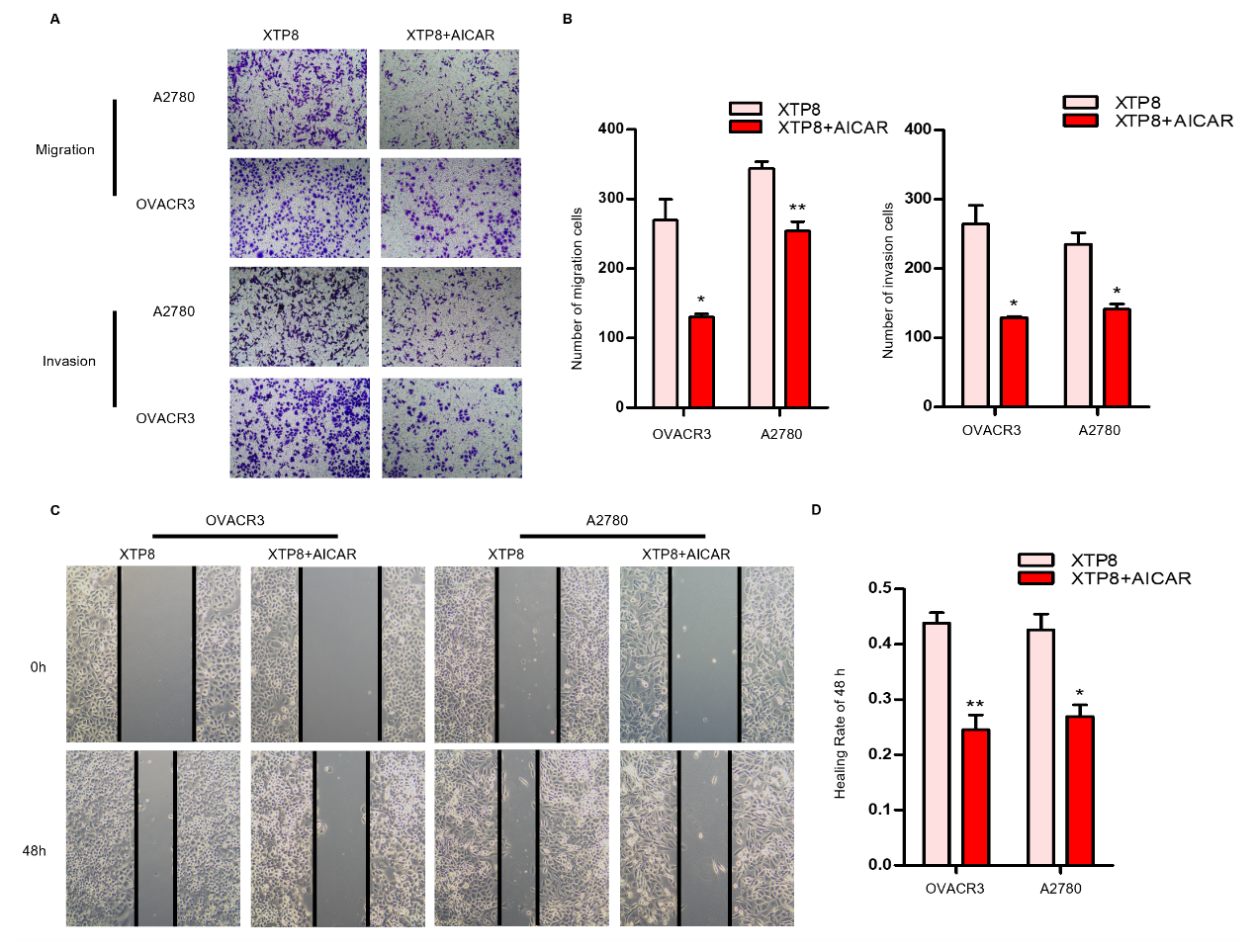

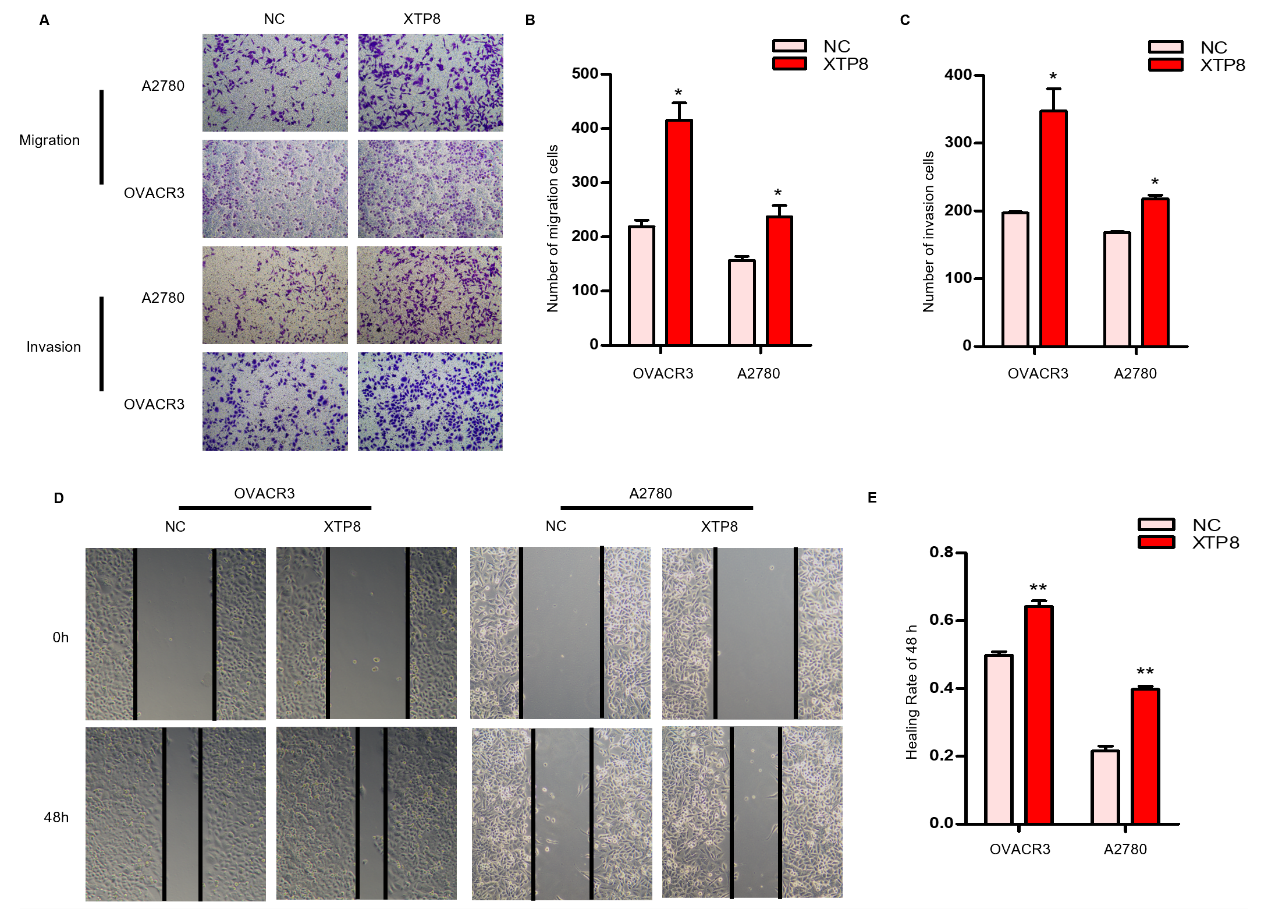
**Fig.S3**


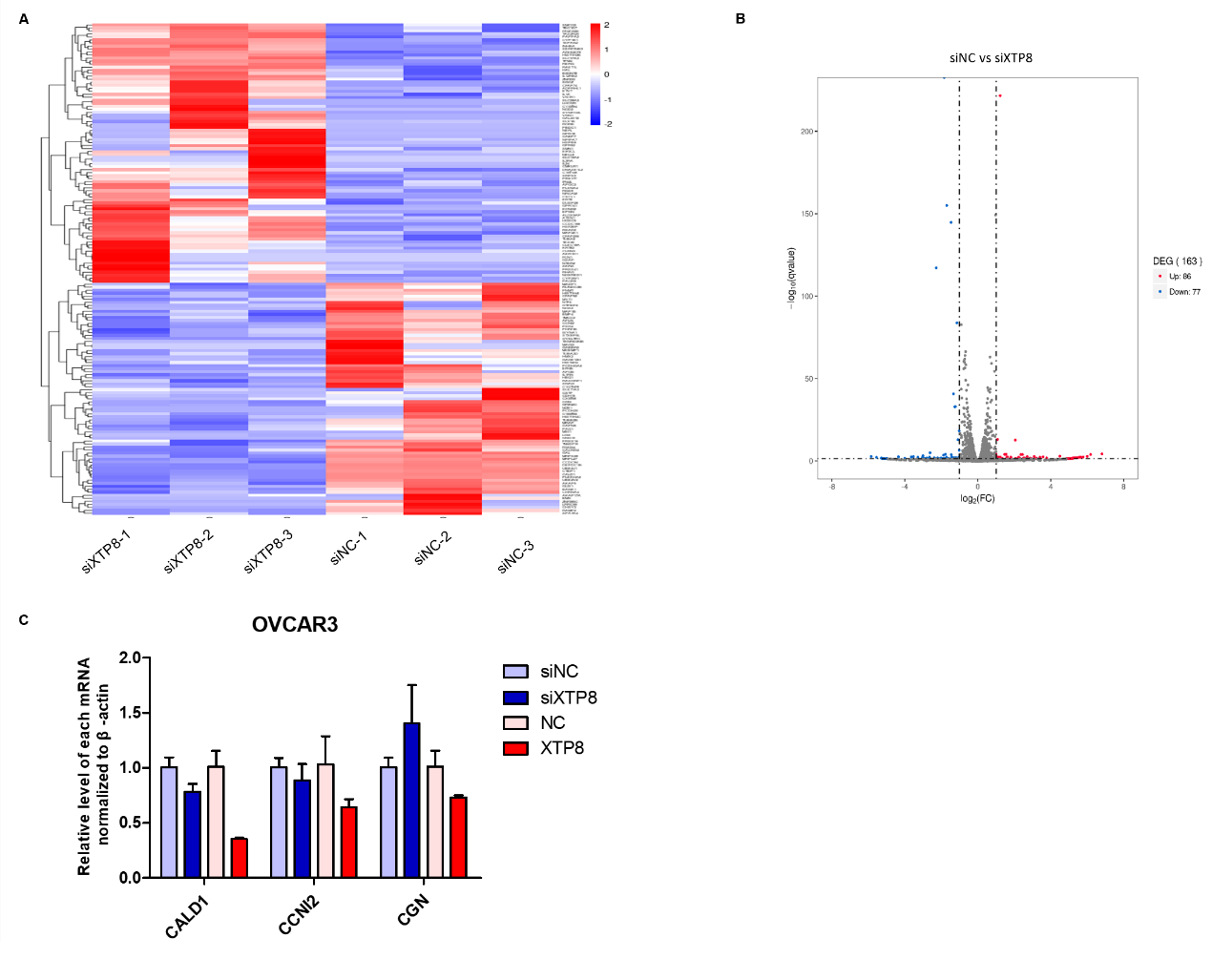
**Fig.S4**

**Supplement Fig. S1** (A and B) Overexpression of XTP8 significantly promotes ovarian cancer cell proliferation. (C and D) Overexpression of XTP8 significantly inhibits ovarian cancer cell apoptosis. (**p*≤0.05, ***p*≤0.01, ****p*≤0.001)

**Supplement Fig. S2** (A, B, and C) Transwell assay shows a significant increase in the number of migrated and invaded cells after overexpression of XTP8. (D) and E Scratch assay demonstrates a significant promotion of wound healing with XTP8 overexpression.

**Supplement Fig. S3** (A and B) Transwell assay reveals that AMPK activator reverses the increased invasion and migration caused by XTP8 overexpression. (C and D) Scratch assay shows that the AMPK activator reverses the accelerated wound healing caused by XTP8 overexpression.

**Supplement Fig. S4** (A and B) Silencing XTP8 results in a total of 163 differentially expressed genes, with 86 upregulated and 77 downregulated. (C) Expression levels of the selected 3 differentially expressed genes.
